# Dehydroevodiamine Alleviates Doxorubicin-Induced Cardiomyocyte Injury by Regulating Neuregulin-1/ErbB Signaling

**DOI:** 10.1155/cdr/5538740

**Published:** 2024-12-07

**Authors:** Song Jie, Guo Wenying, Sun Lebo

**Affiliations:** ^1^Department of Cardiothoracic Surgery, Ningbo Medical Center Lihuili Hospital of Ningbo University, No. 57, Xingning Rd, Ningbo City 315041, Zhejiang Province, China; ^2^Department of Digestive, Ningbo Medical Center Lihuili Hospital of Ningbo University, No. 57, Xingning Rd, Ningbo City 315041, Zhejiang Province, China

**Keywords:** cardiotoxicity, dehydroevodiamine, doxorubicin, NRG1/ErbB pathway

## Abstract

**Background:** Doxorubicin (DOX) is a widely used antitumor drug; however, its use is limited by the risk of serious cardiotoxicity. Dehydroevodiamine (DHE) is a quinazoline alkaloid which has antiarrhythmic effects. The aim of this study was to investigate the protective effect of DHE on doxorubicin-induced cardiotoxicity (DIC) and its potential mechanism.

**Materials and Methods:** Rat H9c2 cardiomyocytes were exposed to DOX for 24 h to establish a DOX-induced cardiomyocyte injury model. DHE and ErbB inhibitor AG1478 were used to treat H9c2 cells to investigate their effects. Cell counting kit-8 (CCK-8) and lactate dehydrogenase (LDH) release assays were used to evaluate cell viability. Flow cytometry and caspase-3 activity assay were used to detect apoptosis. Western blot was used to detect the expression levels of apoptosis-related proteins and neuregulin-1 (NRG1)/ErbB pathway–related proteins. The levels of proinflammatory cytokines and markers of oxidative stress were also detected, respectively. Quantitative polymerase chain reaction (qPCR) was used to detect mRNA expression levels of hub genes.

**Results:** DHE enhanced cardiomyocyte viability and decreased LDH release in a concentration- and time-dependent manner. DHE also significantly inhibited DOX-induced cardiomyocyte apoptosis, inflammation, and oxidative stress. Bioinformatics analysis showed that the protective mechanism of DHE against DIC was related to ErbB signaling pathway. DOX treatment significantly reduced NRG1, p-ErbB2, and p-ErbB4 protein expression levels in cardiomyocytes, while DHE pretreatment reversed this effect. ErbB inhibitor AG1478 reversed the protective effect of DHE on cardiomyocytes.

**Conclusion:** DHE protects cardiomyocytes against DOX by regulating NRG1/ErbB pathway. DHE may be a potential agent for the prevention and treatment of DIC.

## 1. Introduction

Doxorubicin (DOX) is an anthracycline antitumor drug that is widely used to treat lymphoma, sarcoma, breast cancer, and leukemia [1]. However, due to the dose-dependent and cumulative cardiotoxic effects of DOX, its clinical use is limited [2]. DOX can impair the pumping function of the heart, leading to dilated cardiomyopathy and congestive heart failure [3, 4]. A previous report suggests that the incidences of heart failure in patients with cumulative DOX doses of 400, 550, and 700 mg/m^2^ were 3%, 7%, and 18%, respectively [5]. The mechanism of doxorubicin-induced cardiotoxicity (DIC) is complex, involving oxidative stress, mitochondrial dysfunction, and apoptosis [6]. Currently, dexrazoxane is the only drug approved by the US Food and Drug Administration for the treatment of DIC; however, dexrazoxane may reduce the sensitivity of cancer cells to chemotherapy and exacerbate myelosuppression [7]. Therefore, there is an urgent need to develop safe and effective treatment strategies to prevent/alleviate DIC.

Dehydroevodiamine (DHE), a quinazoline alkaloid isolated from evodiamine, has antiarrhythmic effects on ventricular myocytes of guinea pig [8]. DHE has been shown to have good pharmacological activities of organ protection [9]. It has been reported that DHE downregulates the expression of myeloperoxidase, tumor necrosis factor-*α* (TNF-*α*), and interleukin-6 (IL-6) and upregulates the expression of interleukin-10 (IL-10), through the ERK and p38 signaling pathways, thereby improving indomethacin-induced gastric injury [10]. DHE can also reduce neurological dysfunction in mice by ameliorating oxidative stress and apoptosis after traumatic brain injury [11]. In addition, previous studies have shown that DHE has calcium antagonistic activity [12, 13]. However, the role and mechanism of DHE in DIC have not been studied.

Neuregulin-1 (NRG1) is an agonist of the receptor tyrosine kinase of the epidermal growth factor receptor (EGFR) family [14]. It acts mainly through the ErbB family of tyrosine kinase receptors (Erb-b2 receptor tyrosine kinase 2 (ErbB2), ErbB3, and Erb-b2 receptor tyrosine kinase 4 (ErbB4)) [15]. The NRG1/ErbB pathway plays a key role in the development of the cardiovascular system and the maintenance of adult heart function [16, 17]. Notably, the NRG1/ErbB pathway is also involved in the pathogenesis of DIC. It has been reported that DOX treatment inhibits NRG1 expression and inhibits ErbB4 activation, while NRG1 activation alleviates DOX-induced cardiomyocyte apoptosis and improves cardiac function [18, 19]. Overexpression of ErbB2 reduces the base level of reactive oxygen species (ROS), upregulates antioxidant enzymes, and prevents DOX-induced cardiac injury [20]. Therefore, enhancing the activity of the NRG1/ErbB pathway may help to alleviate or prevent DIC.

The aim of this study was to establish a DOX-induced cardiomyocyte injury model in vitro, to evaluate the effect of DHE on DIC, and to explore whether DHE plays a cardioprotective role by regulating the NRG1/ErbB pathway.

## 2. Materials and Methods

### 2.1. Cell Culture

Rat cardiomyocyte line H9c2 was purchased from the Typical Culture Preservation Center, Chinese Academy of Sciences (Shanghai, China). The cells were cultured in 5% CO_2_ at 37°C and in Dulbecco's modified Eagle's medium (DMEM; Invitrogen, Carlsbad, California, United States) supplemented with 10% fetal bovine serum (FBS; Gibco, Carlsbad, California, United States), 100 U/mL penicillin, and 100 *μ*g/mL streptomycin (Gibco, Carlsbad, California, United States).

### 2.2. Cell Grouping and Treatment

According to the previous report, H9c2 cells were treated with 1 *μ*M DOX (GLPBIO, United States, dissolved in dimethyl sulfoxide) for 24 h to establish the DIC model [21]. To evaluate the cytotoxic effects of DHE on H9c2 cells, H9c2 cells were treated with different concentrations (0, 2.5, 5, 10, 20, and 50 *μ*M) of DHE (MedChemExpress, Shanghai, China) for 24 or 48 h. To evaluate the dose-dependent effects of DHE on DOX-induced injury of H9c2 cells, H9c2 cells were grouped into four groups: control group, 1 *μ*M DOX treatment group, 10 *μ*M DHE pretreatment +1 *μ*M DOX treatment group, and 50 *μ*M DHE pretreatment +1 *μ*M DOX treatment group. To evaluate the time-dependent effects of DHE on DOX-induced injury of H9c2 cells, H9c2 cells were grouped into four groups: control group, 1 *μ*M DOX treatment group, 10 *μ*M DHE pretreatment for 24 h +1 *μ*M DOX treatment group, and 10 *μ*M DHE pretreatment for 48 h +1 *μ*M DOX treatment group. To evaluate the effects of DHE on DOX-induced apoptosis, inflammation, and oxidative stress of H9c2 cells, H9c2 cells were grouped into four groups: control group, 1 *μ*M DOX treatment group, 10 *μ*M DHE treatment group, and 1 *μ*M DOX treatment + 10 *μ*M DHE pretreatment group. In addition, to determine whether the ErbB signaling pathway plays a role in the protective effect of DHE on DIC, 10 *μ*M DHE and 10 *μ*M AG1478 (ErbB inhibitor; MedChemExpress, Shanghai, China) were applied to pretreat H9c2 cells for 24 h before DOX treatment.

### 2.3. Cell Viability Assay

Cell counting kit-8 (CCK-8; Beyotime, Shanghai, China) was applied to detect the viability of cardiomyocytes. H9c2 cells were added into 96-well plates at a density of 5 × 10^3^ cells/well and cultured at 37°C for 24 h. After treatment, 10 *μ*L of CCK-8 solution was added to each well and the cells were incubated at 37°C for 2 h. The absorbance value of each well at 450-nm wavelength was then measured with a microplate reader (Bio-Rad, La Jolla, California, United States), and cell viability was calculated.

### 2.4. Determination of Lactate Dehydrogenase (LDH) Release

LDH release was detected using the LDH assay kit (Jiancheng Biotechnology, Nanjing, China). The cells were added into 96-well plates at a density of 1 × 10^4^ cells/well. After treating and processing, the medium was harvested and a microplate reader (Bio-Rad, La Jolla, California, United States) was applied to detect the absorbance value at 530-nm wavelength, and the LDH release was calculated based on the standard curve.

### 2.5. Flow Cytometry

An annexin V-fluorescein isothiocyanate (FITC)/propidium iodide (PI) apoptosis detection kit (Beyotime, Shanghai, China) was used to detect apoptosis of H9c2 cells. In short, H9c2 cells were added in a six-well plate (5 × 10^4^/well) overnight. The treated cells were collected, centrifuged at 1000 × *g* at 4°C for 5 min, and resuspended in 100-*μ*L binding buffer. The cells were then incubated with 5 *μ*L annexin V-FITC and 5 *μ*L PI in the dark at 4°C for 30 min. After the cells were washed with the binding buffer, the apoptosis was assessed using a BD FACSCalibur flow cytometer (BD Biosciences, Franklin Lakes, New Jersey, United States). FlowJo software was used to analyze the results.

### 2.6. Evaluation of Caspase-3 Activity

A caspase-3 activity detection kit (Beyotime, Shanghai, China) was used to detect caspase-3 activity in H9c2 cells. After treatment, H9c2 cells were collected and lyzed with radioimmunoprecipitation assay (RIPA) lysis buffer (Beyotime, Shanghai, China). Protein concentrations were quantified using a bicinchoninic acid (BCA) protein assay kit (Pierce, Rockford, Illinois, United States). Thirty-microgram cell extract was placed in a 96-well plate containing 20 *μ*g acetyl-Asp-Glu-Val-Asp p-nitroanilide (Ac-DEVD-pNA) and incubated at 37°C for 2 h. The release of p-nitroanilide (pNA) was quantified by measuring the absorbance at 405-nm wavelength using a microplate reader (Bio-Rad, La Jolla, California, United States). Caspase-3 activity was calculated as mean absorbance of the test wells/mean absorbance of the control wells × 100%.

### 2.7. Western Blot

An equal amount of protein (40 *μ*g per lane) was separated by sodium dodecyl sulfate–polyacrylamide gel electrophoresis and then transferred to a polyvinylidene difluoride (PVDF) membrane (Millipore, Billerica, Massachusetts, United States). The membranes were then blocked with 5% skim milk at room temperature for 1.5 h. Subsequently, according to the molecular weight, the membranes were cut and incubated with the corresponding primary antibodies overnight at 4°C: anti-B-cell lymphoma 2 (Bcl-2) antibody (ab182858, 1:1000), anti-B-cell lymphoma 2–associated X protein (Bax) antibody (ab182733, 1:1000), anti-NRG1 antibody (ab217805, 1:1000), anti-ErbB2 antibody (ab134182, 1:1000), anti-phospho (p)-ErbB2 antibody (ab316758, 1:1000), anti-ErbB4 antibody (ab137412, 1:1000), anti-p-ErbB4 antibodies (ab61059, 1:1000), and anti-GAPDH antibodies (ab9485, 1:1000). The membranes were then incubated with the secondary antibody (ab6721, 1:5000) at 37°C for 2 h. Finally, the signals of the protein bands were developed using an enhanced chemiluminescence kit (Biozym, Hessisch Oldendorf, Germany) and quantitatively analyzed using Image Lab software (Bio-Rad, Hercules, California, United States). The antibodies used in this study were purchased from Abcam (Shanghai, China).

### 2.8. Enzyme-Linked Immunosorbent Assay (ELISA)

H9c2 cells were added in a six-well plate and cultured at 37°C for 24 h. Next, the cells were collected and centrifuged at 1000 × *g* at 4°C for 10 min, and the supernatant was obtained after centrifugation. According to the manufacturer's protocol, the concentrations of TNF-*α*, IL-1*β*, and IL-6 in the supernatants were measured using the corresponding ELISA kit (Beyotime, Shanghai, China).

### 2.9. ROS Measurement

Fluorescent probe dichlorodihydrofluorescein diacetate (DCFH-DA; Beyotime, Shanghai, China) was utilized to measure the intracellular ROS levels. Briefly, H9c2 cells were added into 96-well plates at a density of 5000 cells per well. After treatment, the cells were incubated with 10-*μ*M DCFH-DA at 37°C for 30 min in the dark. After washing with phosphate-buffered saline (PBS) for three times, fluorescence intensity of each well was measured at excitation wavelength 488 nm and emission wavelength 525 nm using a fluorescence spectrophotometer.

### 2.10. Detection of Malondialdehyde (MDA), Glutathione (GSH), and Superoxide Dismutase (SOD)

In short, H9c2 cells were cultured in a six-well plate at a density of 1 × 10^6^ cells/well. After cell treatment, the cells were washed three times with precooled PBS and harvested. Then, the cells were cleaved with ultrasonic method. According to the manufacturer's instructions, the MDA, GSH, and SOD detection kits (Catalog Nos. S0131, S0053, and S0109; Beyotime, Shanghai, China) were applied to detect the levels of MDA, GSH, and SOD.

### 2.11. Reverse Transcription Quantitative Polymerase Chain Reaction (qPCR)

Total RNA was extracted using a TRIzol kit (Thermo Fisher Scientific, Waltham, Massachusetts, United States). Total RNA was reverse transcribed into cDNA using a reverse transcription kit (Vazyme, Nanjing, China). The ABI 7300 Fast Real-Time PCR system (Applied Biosystems; Thermo Fisher Scientific, Inc., Foster City, California, United States) and SYBR Green PCR kit (Vazyme, Nanjing, China) were used for qPCR. The thermal cycle conditions are as follows: initial denaturation at 95°C for 5 min; then denatured for 1 min at 94°C, annealed for 1 min at 60°C, extended for 1 min at 72°C, a total of 38 cycles; and the final extension at 72°C for 10 min. GAPDH was used as internal reference, and the relative gene expression was calculated by the 2^−△△CT^ method. The sequences of the primers used are shown in Table 1.

### 2.12. Identification of DHE Targets in Treating DIC

The PubChem database (https://pubchem.ncbi.nlm.nih.gov/) was searched to obtain the canonical SMILES file of DHE, and it was imported into the SwissTargetPrediction database (http://www.swisstargetprediction.ch/), to predict potential targets of DHE. In addition, the structured data file (SDF) of DHE was obtained from PubChem and uploaded to the PharmMapper database (http://www.lilab-ecust.cn/pharmmapper/) to obtain additional DHE-related targets. The DIC-related microarray dataset GSE207737 (platform: GPL26962) was retrieved in the GEO database (http://www.ncbi.nlm.nih.gov/geo) with the keyword “doxorubicin cardiotoxicity”. The GSE207737 dataset consisted of three control samples and four DOX-treated samples. Differentially expressed genes (DEGs) were screened using the “limma” R package. The screening criteria for DEGs were *p* < 0.05 and |log2 fold change| > 1. Subsequently, the gene ontology (GO) and Kyoto Encyclopedia of Genes and Genomes (KEGG) pathway enrichment analyses of the target intersection in the intersection were analyzed using the “clusterProfiler” R package. *p* < 0.01 was considered as the enrichment threshold.

### 2.13. Molecular Docking

From PubChem (https://pubchem.ncbi.nlm.nih.gov/), the 2D chemical structures of DHE, as the ligand, were downloaded, and Open Babel software converted the SDF format file to Protein Data Bank (PDB) format file. From PDB (https://www.rcsb.org/) and AlphaFold protein structure database (https://alphafold.ebi.ac.uk/), the structures of the key proteins, as the receptor, were downloaded. AutoDockTools v1.5.7 software was applied to remove water, add polar hydrogen, calculate the charge, and convert all receptors and the ligand files to pdbqt format. Molecular docking analysis was performed using AutoDock Vina v1.1.2. When the binding energy is less than 0, it is considered that receptor and ligand can spontaneously bind and interact with each other [22]. Finally, the docking results were visualized in 3D by PyMOL software.

### 2.14. Statistical Analysis

All experiments were conducted independently in triplicate. SPSS 21.0 software (IBM Corp., Armonk, New York, United States) was used for statistical analysis. Data are expressed as mean ± standard deviation (SD). Multivariate comparisons were made using one-way analysis of variance (ANOVA) and Tukey post hoc tests. *p* < 0.05 was considered statistically significant.

## 3. Results

### 3.1. DHE Enhanced the Viability of DOX-Induced Cardiomyocytes

**Figure 1:**
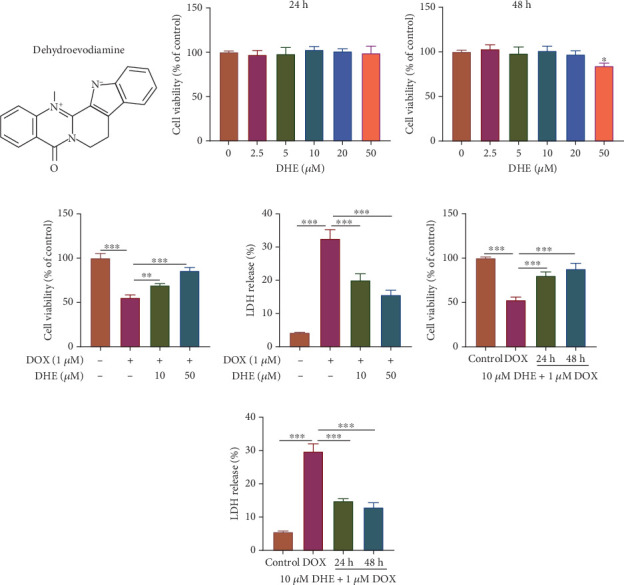
DHE enhances the viability of DOX-induced cardiomyocytes. (a) Chemical structure of DHE. (b, c) H9c2 cardiomyocytes were treated with different concentrations of DHE (0, 2.5, 5, 10, 20, and 50 *μ*M) for 24 h, and cell viability was detected by CCK-8 method. (d, e) H9c2 cells were treated with 1 *μ*M DOX and different concentrations (10 and 50 *μ*M) of DHE for 24 h, and then, cell viability and LDH release were measured using CCK-8 and LDH release methods. (f, g) DOX-induced H9c2 cells were treated with 10 *μ*M DHE for different periods (24 or 48 h), and cell viability and LDH release were measured by CCK-8 and LDH assay kit, respectively. All of the experiments were performed in triplicate. ⁣^∗^, ⁣^∗∗^, and ⁣^∗∗∗^ represent *p* < 0.05, *p* < 0.01, and *p* < 0.001, respectively.

The chemical structure formula of DHE is shown in Figure 1(a). To determine the cytotoxic effects of DHE on cardiomyocytes, H9c2 cells were incubated with different concentrations of DHE for 24 or 48 h. The results of CCK-8 method showed that the survival rate of H9c2 cells was not affected after DHE treatment at different concentrations (0, 2.5, 5, 10, 20, and 50 *μ*M), indicating that DHE dose < 50 *μ*M had no obvious toxic effect on H9c2 cells (Figures 1(b) and 1(c)). Subsequently, CCK-8 and LDH release methods were used to detect the effect of DHE on DOX-induced cardiomyocyte injury. Compared with the control group, DOX significantly reduced the survival rate of H9c2 cells and increased LDH release, while pretreatment of H9c2 cells with DHE (10 and 50 *μ*M) for 24 h significantly improved DOX-induced cell survival and reduced LDH release in a concentration-dependent manner (Figures 1(d) and 1(e)). In addition, pretreatment with 10 *μ*M of DHE for 24 or 48 h significantly increased H9c2 cell viability and reduced LDH release in a time-dependent manner compared to the DOX group (Figures 1(f) and 1(g)).

### 3.2. DHE Represses the Apoptosis of DOX-Induced Cardiomyocytes

**Figure 2:**
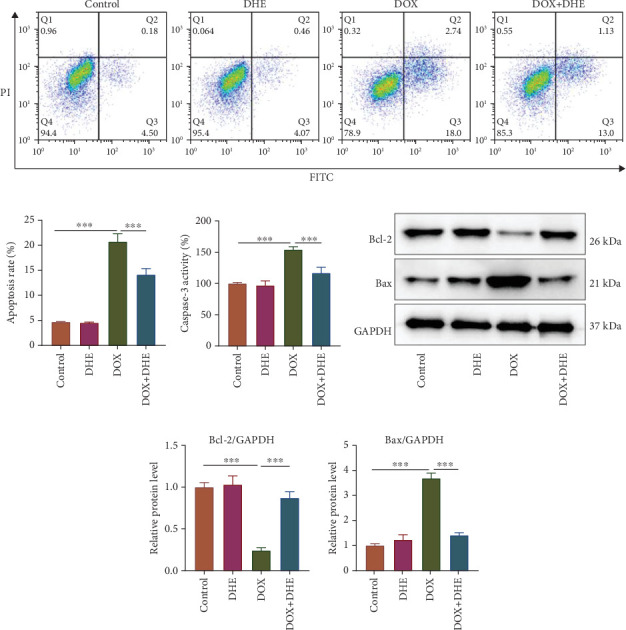
DHE inhibits DOX-induced apoptosis of cardiomyocytes. H9c2 cells were grouped into four groups: control group, 1 *μ*M DOX treatment group, 10 *μ*M DHE treatment group, and 1 *μ*M DOX treatment + 10 *μ*M DHE pretreatment group. (a, b) Flow cytometry was applied to assess apoptosis of H9c2 cells. (c) Caspase-3 activity was detected with caspase-3 detection kit. (d, e) Western blot was used to detect the protein levels of apoptosis-related proteins Bcl-2 and Bax. All of the experiments were performed in triplicate. ⁣^∗∗∗^ represents *p* < 0.001.

Next, we investigated the effect of DHE on DOX-induced apoptosis of cardiomyocytes. Flow cytometry analysis showed that DHE pretreatment significantly reduced DOX-induced apoptosis of H9c2 cardiomyocytes (Figures 2(a) and 2(b)). In DOX-induced H9c2 cells, a significant increase in caspase-3 activity was revealed, while DHE pretreatment decreased caspase-3 activity (Figure 2(c)). In addition, Western blot results showed that compared with the control group, the protein expression level of Bcl-2 in DOX-treated H9c2 cells was significantly reduced, while the protein expression level of Bax was significantly increased; however, DHE pretreatment significantly upregulated Bcl-2 expression and decreased Bax expression in DOX-induced H9c2 cells (Figures 2(d) and 2(e)). These results indicated that DHE could inhibit DOX-induced apoptosis of cardiomyocytes.

### 3.3. DHE Alleviates DOX-Induced Inflammation and Oxidative Stress in Cardiomyocytes

**Figure 3:**
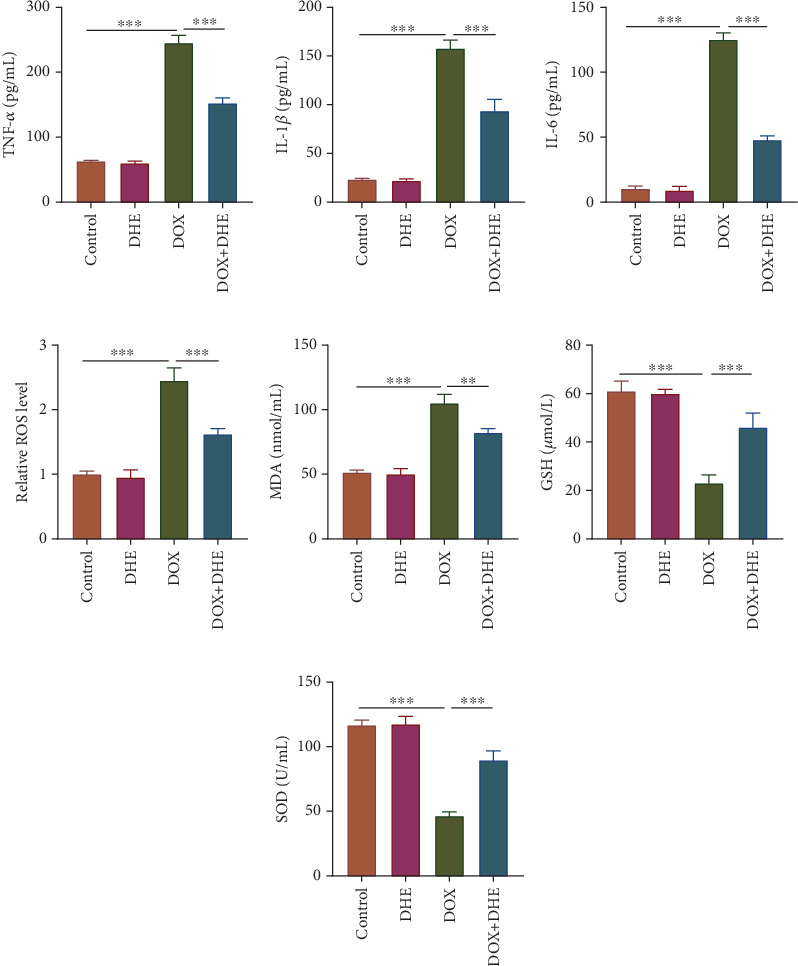
DHE inhibits DOX-induced inflammation and oxidative stress in cardiomyocytes. H9c2 cells were grouped into four groups: control group, 1 *μ*M DOX treatment group, 10 *μ*M DHE treatment group, and 1 *μ*M DOX treatment + 10 *μ*M DHE pretreatment group. (a–c) The levels of proinflammatory cytokines TNF-*α*, IL-1*β*, and IL-6 were detected by ELISA. (d) The intracellular ROS production was detected by DCFH-DA probe. (e–g) The levels of MDA, GSH, and SOD in H9c2 cells were detected. All of the experiments were performed in triplicate. ⁣^∗∗^ and ⁣^∗∗∗^ represent *p* < 0.01 and *p* < 0.001, respectively.

In order to explore whether the protective effect of DHE in DIC was related to regulation of inflammation and oxidative stress, the relevant markers of inflammation and oxidative stress were detected. ELISA showed that DOX significantly increased the levels of inflammatory cytokines TNF-*α*, IL-1*β*, and IL-6 in H9c2 cardiomyocytes compared with the control group; however, DHE pretreatment reversed this effect (Figures 3(a), 3(b), and 3(c)). In addition, DOX treatment resulted in increased ROS level in H9c2 cells, while DHE pretreatment inhibited DOX-induced ROS production (Figure 3(d)). Meanwhile, the levels of MDA, SOD, and GSH in the supernatant of H9c2 cells were also detected. Compared with the control group, MDA content in the supernatant of H9c2 cells in DOX group was increased, while SOD and GSH levels were decreased (Figures 3(e), 3(f), and 3(g)); after DHE treatment, MDA content decreased, while SOD and GSH levels were increased (Figures 3(e), 3(f), and 3(g)). These results suggest that DHE can reduce DOX-induced cardiomyocyte damage by inhibiting inflammatory response and oxidative stress.

### 3.4. Identification of the Crucial Targets of DHE in DIC Treatment

**Figure 4:**
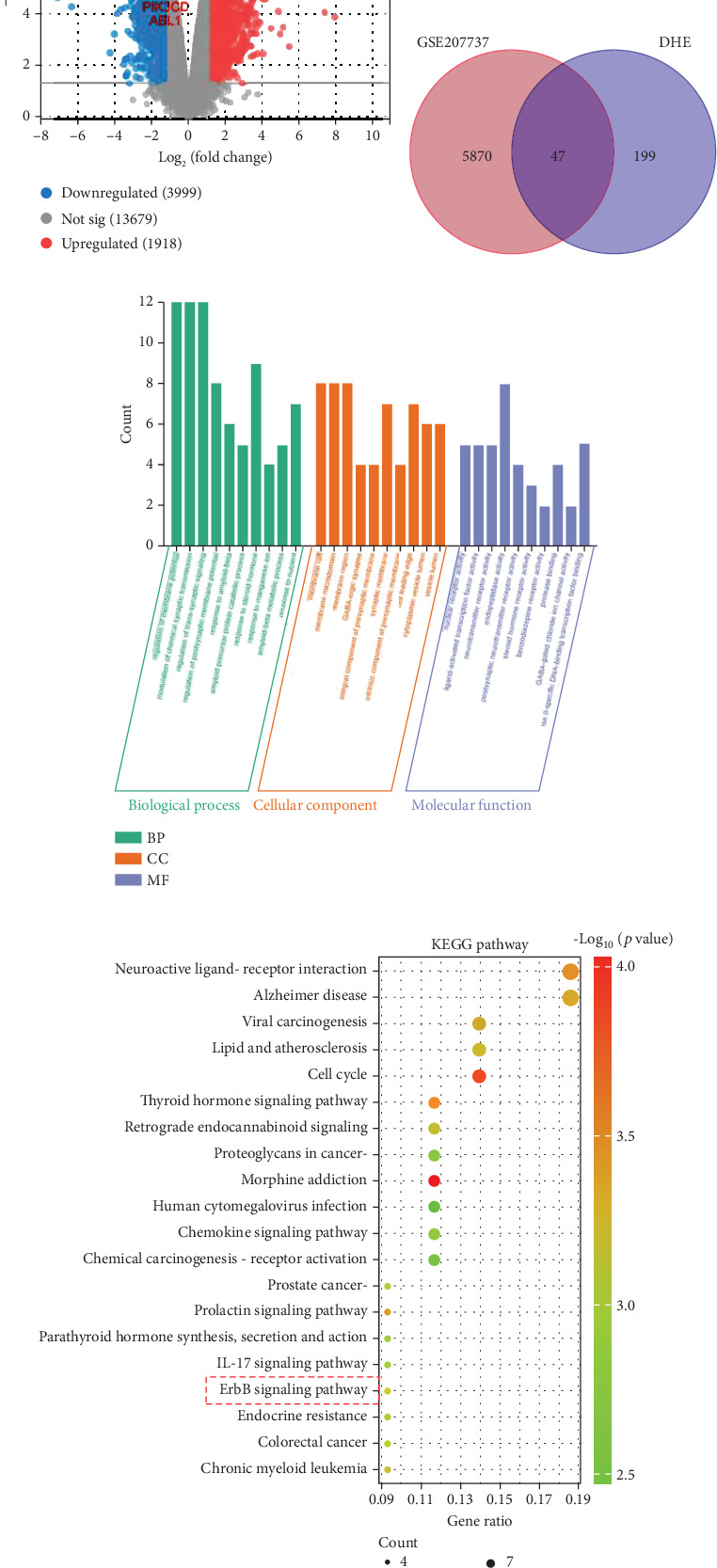
Functional enrichment analysis of DHE targets in treating DIC. (a) Volcano map for the differentially expressed genes of the GSE207737 dataset. *p* < 0.05 and |log2 fold change| > 1 are the threshold. Red dots indicate upregulated genes, blue dots downregulated genes, and gray genes with insignificant differences. (b) Venn diagram of DHE-related targets and DIC-related targets. (c) Histogram of GO analysis of DHE targets in DIC treatment. Biological process (BP) is marked by dark cyan, cellular component (CC) is marked by sienna, and molecular function (MF) is marked by steel blue. (d) Bubble map of KEGG pathway enrichment analysis of DHE targets in DIC treatment. The bubble size represents count, and the bubble color represents the *p* value.

One hundred and three and 150 DHE targets were collected from SwissTargetPrediction and PharmMapper databases, respectively. After integration and removing the duplicates, 246 DHE targets were obtained. Five thousand nine hundred and seventeen DIC-related DEGs were identified from the GSE207737 dataset, including 1918 upregulated genes and 3999 downregulated genes (Figure 4(a)). With a Venn diagram, a total of 47 candidate targets of DHE in DIC treatment were obtained (Figure 4(b)). Subsequently, these candidate targets were subjected to GO and KEGG enrichment analysis. The results of GO enrichment showed that 681 GO terms were significantly enriched, including 595 biological processes, 45 cellular components, and 41 molecular functions. The top five biological processes are regulation of membrane potential, modulation of chemical synaptic transmission, regulation of trans-synaptic signaling, regulation of postsynaptic membrane potential, and response to amyloid beta (Figure 4(c)); the top five cellular components were membrane raft, membrane microdomain, membrane region, GABAergic synapse, and integral component of presynaptic membrane (Figure 4(c)); the top five molecular functions are nuclear receptor activity, ligand-activated transcription factor activity, neurotransmitter receptor activity, endopeptidase activity, and postsynaptic neurotransmitter receptor activity (Figure 4(c)). In addition, KEGG pathway enrichment analysis showed that a total of 26 KEGG pathways were associated with the mechanism of action of DHE in treating DIC, including the ErbB signaling pathway (Table 2). The top 10 pathways were morphine addiction, cell cycle, thyroid hormone signaling pathway, neuroactive ligand–receptor interaction, prolactin signaling pathway, viral carcinogenesis, Alzheimer disease, chronic myeloid leukemia, lipid and atherosclerosis, and retrograde endocannabinoid signaling (Figure 4(d)).

### 3.5. Molecular Docking Analysis

**Figure 5:**
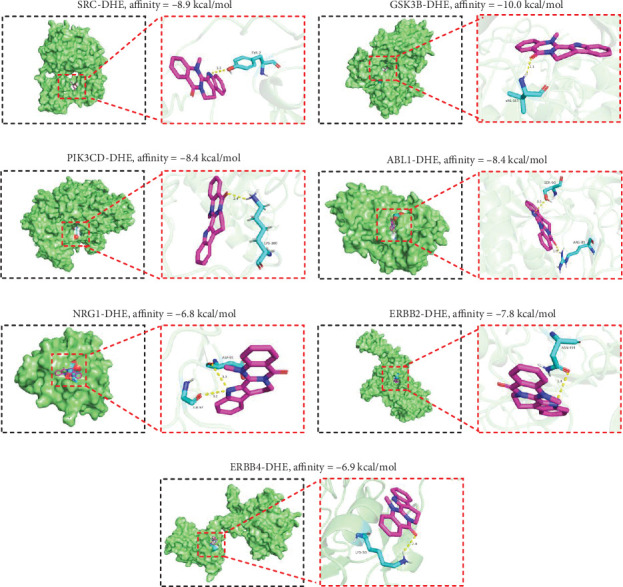
Molecular docking analysis. Molecular docking diagram of DHE with (a) SRC, (b) GSK3B, (c) PIK3CD, (d) ABL1, (e) NRG1, (f) ErbB2, and (g) ErbB4 proteins. Light blue indicates amino acid residues surrounding the binding bag, purple indicates DHE, green indicates macromolecules, and yellow dashed lines indicate hydrogen bonding.

The NRG1/ErbB pathway plays a key role in regulating cardiac pathophysiology [18]. Interestingly, as mentioned above, the ErbB signaling pathway is also involved in the mechanism of action of DHE in DIC treatment. Therefore, the four targets of DHE in the ErbB signaling pathway, including SRC proto-oncogene, nonreceptor tyrosine kinase (SRC), glycogen synthase kinase 3 beta (GSK3B), phosphatidylinositol-4,5-bisphosphate 3-kinase catalytic subunit delta (PIK3CD), and ABL proto-oncogene 1, nonreceptor tyrosine kinase (ABL1), were chosen for further investigation. The binding energies between DHE and SRC, GSK3B, PIK3CD, ABL1, NRG1, and ErbB2 proteins ranged from −10.0 to −6.8 kcal/mol. The 3D conformations of these complexes with the lowest binding energy are shown (Figure 5). DHE formed a hydrogen bond with TRY-7, the amino acid residue of SRC protein (PDB ID 4K11), with a binding affinity of −8.9 kcal/mol (Figure 5(a)). DHE formed a hydrogen bond with VAL-105, the amino acid residue of GSK3B protein (PDB ID 8AV1), with a binding affinity of −10.0 kcal/mol (Figure 5(b)). DHE formed a hydrogen bond with LYS-380, the amino acid residue of PIK3CD protein (PDB ID 8BCY), with a binding affinity of −8.4 kcal/mol (Figure 5(c)). DHE formed two hydrogen bonds with amino acid residues SER-93 and ARG-89 of ABL1 protein (PDB ID 5MO4), with binding affinity = −8.4 kcal/mol (Figure 5(d)). DHE formed two hydrogen bonds with the amino acid residues ASP-95 and GLY-97 of NRG1 protein, and the binding affinity is −6.8 kcal/mol (Figure 5(e)). DHE formed a hydrogen bond with ASN-454, the amino acid residue of ErbB2 protein (PDB ID 5MY6), and the binding affinity is −7.8 kcal/mol (Figure 5(f)). DHE formed a hydrogen bond with LYS-363, the amino acid residue of ErbB4 protein (PDB ID 3U2P), and the binding affinity is −6.9 kcal/mol (Figure 5(g)). Taken together, these results suggest that DHE has stable binding capacity to NRG1/ErbB pathway-related targets, suggesting that DHE may play a protective role in DOX-mediated myocardial injury through the NRG1/ErbB pathway.

### 3.6. DHE Regulates the NRG1/ErbB Pathway in Cardiomyocytes Treated With DOX

**Figure 6:**
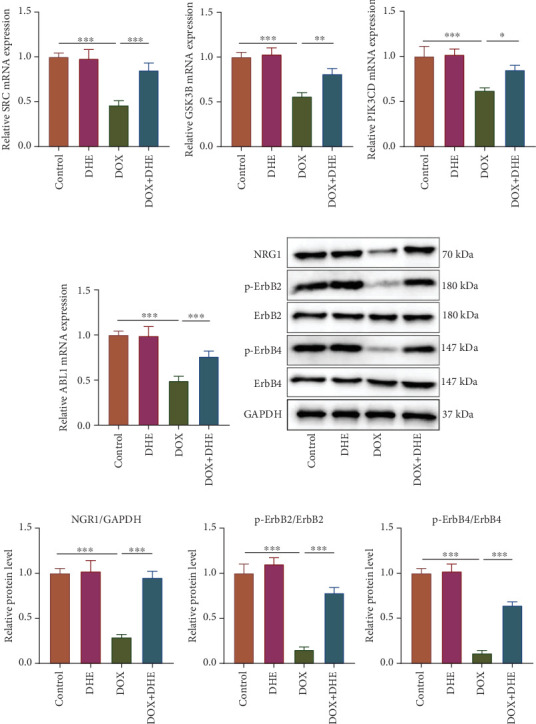
Effect of DHE on the NRG1/ErbB2 pathway in DOX-induced cardiomyocytes. H9c2 cells were grouped into four groups: control group, 1 *μ*M DOX treatment group, 10 *μ*M DHE treatment group, and 1 *μ*M DOX treatment + 10 *μ*M DHE pretreatment group. (a–d) qPCR was used to detect the mRNA expression levels of SRC, GSK3B, PIK3CD, and ABL1. (e, f) Protein expression levels of NRG1, p-ErbB2, and p-ErbB4 were detected by Western blot. All of the experiments were performed in triplicate. ⁣^∗^, ⁣^∗∗^, and ⁣^∗∗∗^ represent *p* < 0.05, *p* < 0.01, and *p* < 0.001, respectively.

In order to verify whether the protective effect of DHE on DIC is related to the NRG1/ErbB2 pathway, qPCR was used to detect mRNA expression levels of crucial targets (SRC, GSK3B, PIK3CD, and ABL1) in the ErbB signaling pathway. mRNA expression levels of SRC, GSK3B, PIK3CD, and ABL1 were significantly inhibited in H9c2 cells in the DOX group compared to the control group, and DHE pretreatment significantly reversed these effects (Figures 6(a), 6(b), 6(c), and 6(d)). Western blot showed that DOX treatment significantly reduced the protein levels of NRG1, p-ErbB2, and p-ErbB4, while DHE pretreatment significantly upregulated the protein levels of NRG1, p-ErbB2, and p-ErbB4 in DOX-induced H9c2 cells (Figures 6(e) and 6(f)).

### 3.7. Inhibition of the ErbB Pathway Limited the Protective Effect of DHE on DOX-Induced Cardiomyocyte Injury

**Figure 7:**
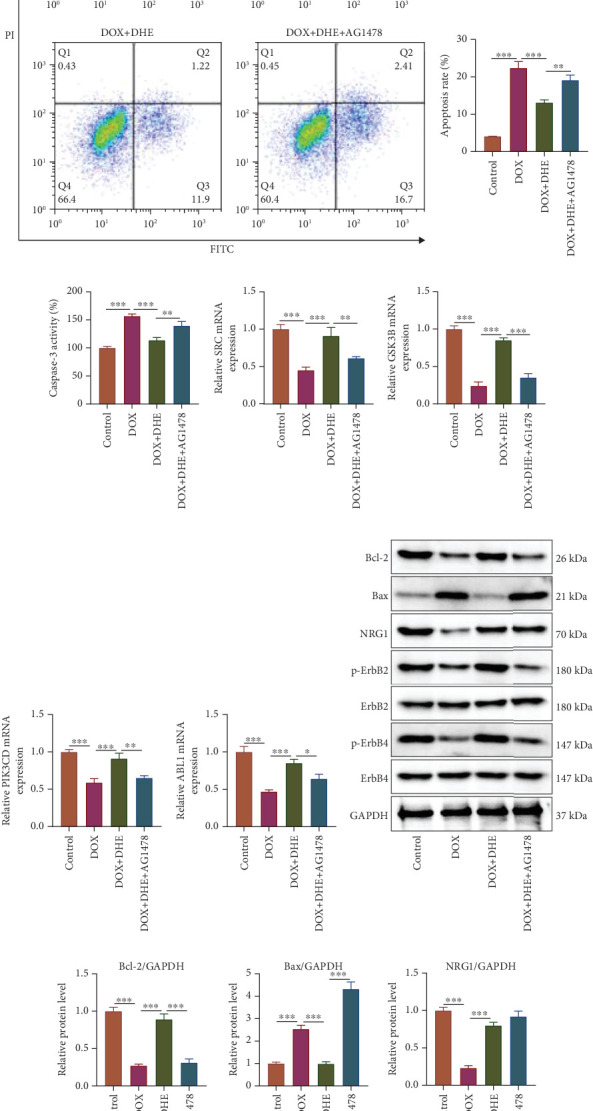
Effects of ErbB inhibitor AG1478 on DOX-induced injury of H9c2 cells after DHE treatment. H9c2 cells were grouped into four groups: control group, 1 *μ*M DOX treatment group, 1 *μ*M DOX treatment + 10 *μ*M DHE pretreatment group, and 1 *μ*M DOX treatment + 10 *μ*M DHE pretreatment + 10 *μ*M AG1478 pretreatment group. (a, b) The cell viability and LDH release of H9c2 cells were measured by CCK-8 assay and LDH release method. (c, d) Apoptosis of H9c2 cells was assessed by flow cytometry. (e) Caspase-3 activity was detected with caspase-3 detection kit. (f–i) qPCR was used to detect the mRNA expression levels of SRC, GSK3B, PIK3CD, and ABL1. (j, k) Western blot was used to detect protein expression levels of Bcl-2, Bax, NRG1, p-ErbB2, and p-ErbB4. All of the experiments were performed in triplicate. ⁣^∗^, ⁣^∗∗^, and ⁣^∗∗∗^ represent *p* < 0.05, *p* < 0.01, and *p* < 0.001, respectively.

Next, we treated DOX-induced H9c2 cardiomyocytes with DHE and ErbB inhibitor AG1478 for 24 h. DHE significantly enhanced DOX-induced H9c2 cardiomyocyte viability and reduced LDH release, while AG1478 treatment significantly reversed this effect (Figures 7(a) and 7(b)). Flow cytometry showed that AG1478 reversed the inhibitory effect of DHE on DOX-induced apoptosis of cardiomyocytes (Figures 7(c) and 7(d)). Consistently, DHE significantly reduced caspase-3 activity in DOX-induced H9c2 cells, while AG1478 significantly reversed this effect (Figure 7(e)). In addition, qPCR results showed that AG1478 treatment significantly attenuated the upregulation effect of DHE on mRNA expression levels of SRC, GSK3B, PIK3CD, and ABL1 in DOX-induced H9c2 cells (Figures 7(f), 7(g), 7(h), and 7(i)). Western blot showed that compared with the DOX group, Bax protein was significantly downregulated after DHE treatment, while Bcl-2, NRG1, p-ErbB2, and p-ErbB4 proteins were significantly upregulated (Figures 7(j) and 7(k)); after AG1478 administration, Bax protein expression was upregulated, while Bcl-2, p-ErbB2, and p-ErbB4 protein expression levels were significantly downregulated, and NRG1 protein expression level was not significantly affected (Figures 7(j) and 7(k)).

### 3.8. Inhibition of ErbB Reversed the Inhibitory Effect of DHE on DOX-Induced Inflammation and Oxidative Stress in Cardiomyocytes

**Figure 8:**
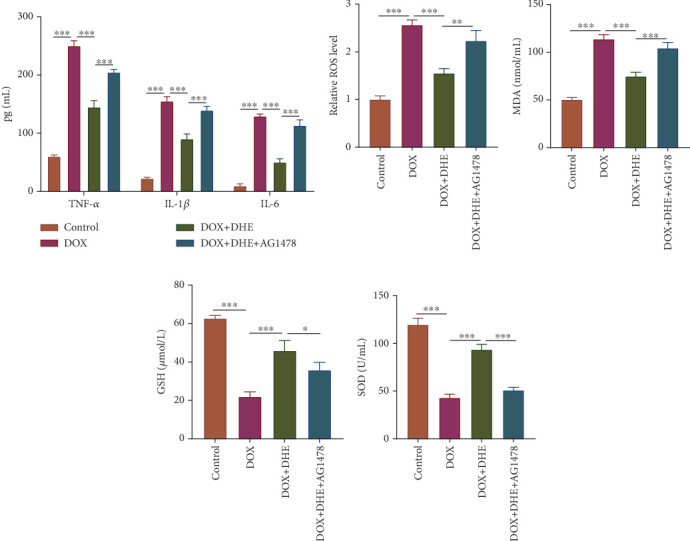
Effects of ErbB inhibition on DOX-induced inflammation and oxidative stress in H9c2 cells after DHE treatment. H9c2 cells were grouped into four groups: control group, 1 *μ*M DOX treatment group, 1 *μ*M DOX treatment + 10 *μ*M DHE pretreatment group, and 1 *μ*M DOX treatment + 10 *μ*M DHE pretreatment + 10 *μ*M AG1478 pretreatment group. (a) The levels of proinflammatory cytokines TNF-*α*, IL-1*β*, and IL-6 were detected by ELISA. (b) The intracellular ROS levels were detected by DCFH-DA probe. (c–e) The levels of MDA, GSH, and SOD were detected by the corresponding detection kit. All of the experiments were performed in triplicate. ⁣^∗^, ⁣^∗∗^, and ⁣^∗∗∗^ represent *p* < 0.05, *p* < 0.01, and *p* < 0.001, respectively.

Next, we investigated the effects of inhibition of the ErbB pathway on the anti-inflammatory and antioxidant activities of DHE. ELISA showed that DHE reduced the levels of TNF-*α*, IL-1*β*, and IL-6 in DOX-induced H9c2 cells, while AG1478 treatment limited the anti-inflammatory effects of DHE (Figure 8(a)). In addition, compared with the DOX group, ROS and MDA levels were decreased and GSH and SOD levels were increased after DHE treatment, while AG1478 treatment significantly weakened DHE's antioxidant effects (Figures 8(b), 8(c), 8(d), and 8(e)). Overall, these data suggest that the ErbB pathway mediates the protective effect of DHE on DOX-induced cardiomyocyte injury.

## 4. Discussion

DOX is a widely used antitumor chemotherapy drug, but it has high risk of cardiotoxicity [2–5]. So far, there is a lack of effective treatment to protect this side effect [23, 24]. DHE, as a quinazoline alkaloid, has great potential in treating various diseases, such as chronic atrophic gastritis, Alzheimer disease, cancer, and cardiovascular disease [25–28]. Its broad therapeutic effect is due to a variety of biological activities, such as anti-inflammatory, antioxidant, antithrombotic, anticholinesterase, antiamnesia, anticancer, neuroprotective, and vasodilatory activities [9]. This study demonstrated for the first time that DHE can effectively attenuate DOX-induced cardiomyocyte injury by reducing apoptosis, inhibiting inflammation and oxidative stress, and the mechanism is associated with the regulation of the NRG1/ErbB pathway.

DOX-induced myocardial injury is accompanied by excessive apoptosis. Cardiomyocyte apoptosis is an important factor leading to myocardial dysfunction and heart failure [29, 30]. As an effector enzyme, caspase-3 is a major actor responsible for promoting apoptosis [31]. Bcl-2 and Bax are major members of the Bcl-2 protein family involved in mitochondria-mediated apoptosis [32]. It has been reported that DOX induces cardiomyocyte apoptosis through the endogenous mitochondrial pathway by regulating the expression of Bax, Bcl-2, and caspase-3 [33]. In this study, we found that DOX treatment resulted in a decrease in H9c2 cell viability and an increase in LDH release. DOX could also induce the apoptosis of H9c2 cells, increase the activity of caspase-3 and the level of Bax protein, and decrease the level of Bcl-2 protein, which is consistent with previous studies [21, 30]. Notably, DHE pretreatment significantly reversed these effects, suggesting that DHE could inhibit DOX-induced cytotoxicity and apoptosis of cardiomyocytes.

Oxidative stress and inflammation have been confirmed to be the main mechanisms of DIC [24]. DOX increases the production of free radicals such as ROS [34]. Under normal physiological conditions, free radicals can be cleared by antioxidant enzymes such as SOD and GSH. However, DOX treatment leads to an imbalance between oxidizer production and antioxidant defense, thereby damaging cell membranes, proteins, and DNA and triggering inflammatory responses [33, 35, 36]. Inflammatory response exacerbates myocardial injury, leading to further damage and death of cardiomyocytes by activating immune cells and releasing inflammatory mediators such as TNF-*α* and IL-6 [37]. In DOX-induced H9c2 cardiomyocytes, the levels of ROS, MDA, and proinflammatory cytokines (TNF-*α*, IL-6, and IL-1*β*) were significantly increased, while the activities of SOD and GSH were inhibited [38–40], which was consistent with our findings. Importantly, this study found that DHE pretreatment significantly reduced the levels of ROS and MDA, thereby enhancing the resistance of H9c2 cardiomyocytes to oxidative stress. At the same time, DHE also increased the activity of antioxidant enzymes such as SOD and GSH, which further confirmed its antioxidant effect. In addition, we investigated the effect of DHE on the inflammatory response of cardiomyocytes. It was found that DHE significantly reduced the levels of inflammatory factors such as TNF-*α*, IL-6, and IL-1*β* in DOX-induced H9c2 cardiomyocytes. Taken together, these findings suggest that DHE can effectively inhibit DOX-induced oxidative stress and inflammatory responses in cardiomyocytes.

The NRG1/ErbB pathway is essential for maintaining adult heart function. Activation of the NRG1/ErbB2 pathway in zebrafish or mouse hearts has been reported to induce dedifferentiation and proliferation of cardiomyocytes [17]. Importantly, DOX-induced cardiovascular disease has been shown to be associated with NRG1/ErbB pathway inhibition. Overexpression of ErbB2 has been reported to reduce the basal level of ROS, upregulate antioxidant enzymes, and prevent DOX-induced cardiac injury [20]. Upregulation of microRNA-146a can aggravate DOX-induced acute cardiotoxicity by targeting the NRG1/ErbB4 pathway [41]. It is worth mentioning that KEGG analysis showed that candidate targets for DHE treatment of DIC were significantly enriched in the ErbB signaling pathway. Molecular docking analysis showed that DHE could stably bind to NRG1, ErbB2, and ErbB4 proteins. At the same time, DHE also has strong binding activity with ErbB signaling pathway–related targets such as SRC, GSK3B, PIK3CD, and ABL1, suggesting that the therapeutic effect of DHE may be related to the NRG1/ErbB pathway. Studies have shown that overexpression of SRC can reduce DOX-induced cytotoxicity [42]. GSK3B is a serine/threonine kinase and a known regulator of cardiomyocyte survival [43]. Cariporide has been reported to reduce DIC in rats by activating the protein kinase B (Akt)/GSK3B survival signaling pathway [38]. PIK3CD is part of the phosphatidylinositol-3-kinase (PI3K)/Akt signaling pathway, which is critical in cell growth, survival, and metabolism. Studies have shown that capsaicin can inhibit DOX-induced apoptosis of cardiomyocytes by activating the PI3K/Akt signaling pathway [44]. In addition, studies have confirmed that ABL1 tyrosine kinase inhibitors (ponatinib) exert cardiotoxicity through off-target effects on cardiomyocyte prosurvival signaling pathways Akt and ERK [45]. In this study, DOX was found to downregulate the mRNA expression of SRC, GSK3B, PIK3CD, and ABL1, which was consistent with the results of GEO dataset analysis. DOX also reduces NRG1, p-ErbB2, and p-ErbB4 protein levels in H9c2 cardiomyocytes. However, DHE pretreatment significantly reversed the effects of DOX on the NRG1/ErbB pathway. In order to further verify the potential role of the NRG1/ErbB pathway in DHE anti-DIC, we studied the effect of the inhibitor AG1478 of the ErbB pathway on DHE treatment of DIC. We found that AG1478 treatment significantly reversed the protective effect of DHE on DOX-induced H9c2 cardiomyocyte injury. These findings suggest that DHE may play a therapeutic role in DOX-induced cardiomyocyte injury by regulating the NRG1/ErbB pathway.

## 5. Conclusion

DHE can potentially relieve DIC by improving cardiomyocyte viability, reducing apoptosis, and inhibiting the production of inflammatory cytokines and oxidative stress factors. DHE may play a protective role by regulating the NRG1/ErbB pathway. These results provide a basis for DHE as a potential treatment option for DIC. In order to further verify the effect of DHE on DIC, in vivo experiments are needed, and additionally, the effects of DHE on cancer cells should be explored in the following work.

## Figures and Tables

**Table 1:** Sequences of the primers used for RT-qPCR.

**Gene**	**Primers**
SRC	Forward: 5⁣′-AGTCCCCTGGCTCGGTTAG-3⁣′
	Reverse: 5⁣′-TGTCATGGCTACACAGGTCG-3⁣′
GSK3B	Forward: 5⁣′-AGCTCTGATTGGCCACTGTC-3⁣′
	Reverse: 5⁣′-TCCTTCCTTTGTCACTCGGC-3⁣′
PIK3CD	Forward: 5⁣′-CCTGCTGGAGGAAGTCTGTG-3⁣′
	Reverse: 5⁣′-TTCAGCTGGATGTTGGCGAT-3⁣′
ABL1	Forward: 5⁣′-TGAAGTTGGTGGGCTGCAAA-3⁣′
	Reverse: 5⁣′-GACCCGGAGCTTTTCACCTT-3⁣′
GAPDH	Forward: 5⁣′-CTTCTCCTGCAGCCTCGT-3⁣′
	Reverse: 5⁣′-ACTGTGCCGTTGAATTTGCC-3⁣′

**Table 2:** The results of KEGG pathway enrichment related to doxorubicin-induced cardiotoxicity treated with dehydroevodiamine.

**ID**	**Description**	**Gene ratio**	**p** ** value**	**Gene ID**	**Count**
hsa05032	Morphine addiction	5/43	7.30468e − 05	GRK2/GRK6/GABRB3/PDE4A/GABRG2	5
hsa04110	Cell cycle	6/43	0.000104411	TGFB2/PRKDC/MDM2/GSK3B/HDAC8/ABL1	6
hsa04919	Thyroid hormone signaling pathway	5/43	0.000291348	RXRB/SRC/MDM2/GSK3B/PIK3CD	5
hsa04080	Neuroactive ligand–receptor interaction	8/43	0.00033623	TACR2/P2RX3/GRM5/GABRB3/TSPO/CNR1/GABRG2/DRD2	8
hsa04917	Prolactin signaling pathway	4/43	0.000371761	ESR2/SRC/GSK3B/PIK3CD	4
hsa05203	Viral carcinogenesis	6/43	0.000429901	PKM/SRC/MDM2/CASP3/PIK3CD/HDAC8	6
hsa05010	Alzheimer disease	8/43	0.000505048	PSENEN/APP/BACE1/GRM5/APH1A/CASP3/GSK3B/PIK3CD	8
hsa05220	Chronic myeloid leukemia	4/43	0.000506956	TGFB2/MDM2/PIK3CD/ABL1	4
hsa05417	Lipid and atherosclerosis	6/43	0.000567522	RXRB/SRC/CASP3/GSK3B/PIK3CD/MMP3	6
hsa04723	Retrograde endocannabinoid signaling	5/43	0.000729667	MGLL/GRM5/GABRB3/CNR1/GABRG2	5
hsa04012	ErbB signaling pathway	4/43	0.000770709	SRC/GSK3B/PIK3CD/ABL1	4
hsa05210	Colorectal cancer	4/43	0.000805014	TGFB2/CASP3/GSK3B/PIK3CD	4
hsa04657	IL-17 signaling pathway	4/43	0.00111941	CASP3/GSK3B/MMP3/MMP13	4
hsa05215	Prostate cancer	4/43	0.001256835	MDM2/GSK3B/PIK3CD/MMP3	4
hsa01522	Endocrine resistance	4/43	0.001305158	ESR2/SRC/MDM2/PIK3CD	4
hsa04928	Parathyroid hormone synthesis, secretion, and action	4/43	0.002265915	RXRB/VDR/PDE4A/MMP13	4
hsa04062	Chemokine signaling pathway	5/43	0.002308495	GRK2/SRC/GRK6/GSK3B/PIK3CD	5
hsa05205	Proteoglycans in cancer	5/43	0.002936525	TGFB2/SRC/MDM2/CASP3/PIK3CD	5
hsa05207	Chemical carcinogenesis–receptor activation	5/43	0.003680394	ESR2/RXRB/SRC/VDR/PIK3CD	5
hsa05163	Human cytomegalovirus infection	5/43	0.004551567	SRC/MDM2/CASP3/GSK3B/PIK3CD	5
hsa01524	Platinum drug resistance	3/43	0.005669651	MDM2/CASP3/PIK3CD	3
hsa05226	Gastric cancer	4/43	0.005873487	TGFB2/RXRB/GSK3B/PIK3CD	4
hsa01521	EGFR tyrosine kinase inhibitor resistance	3/43	0.006783446	SRC/GSK3B/PIK3CD	3
hsa05161	Hepatitis B	4/43	0.007851022	TGFB2/SRC/CASP3/PIK3CD	4
hsa04727	GABAergic synapse	3/43	0.009097079	SRC/GABRB3/GABRG2	3
hsa04540	Gap junction	3/43	0.009958864	SRC/GRM5/DRD2	3

## Data Availability

The data that support the findings of this study are available from the corresponding author upon reasonable request.
